# Determinants of Disability in Multiple Sclerosis: An Immunological and MRI Study

**DOI:** 10.1155/2014/875768

**Published:** 2014-04-09

**Authors:** Paola Tortorella, Maria Marcella Laganà, Marina Saresella, Eleonora Tavazzi, Maria Giulia Preti, Cristian Ricci, Francesca Baglio, Ivana Marventano, Federica Piancone, Giuseppe Baselli, Pietro Cecconi, Domenico Caputo, Mario Clerici, Marco Rovaris

**Affiliations:** ^1^Unit of Motor Neurorehabilitation, Multiple Sclerosis Center, Fondazione Don Gnocchi, IRCCS Santa Maria Nascente, Via Capecelatro 66, 20148 Milan, Italy; ^2^MR Research Laboratory, Fondazione Don Gnocchi, IRCCS Santa Maria Nascente, Via Capecelatro 66, 20148 Milan, Italy; ^3^Immunology Research Laboratory, Fondazione Don Gnocchi, IRCCS Santa Maria Nascente, Via Capecelatro 66, 20148 Milan, Italy; ^4^Bioengineering Department, Politecnico di Milano, Piazza Leonardo da Vinci, 20133 Milan, Italy; ^5^Epidemiology and Statistics Unit, Fondazione Don Gnocchi, IRCCS Santa Maria Nascente, Via Capecelatro 66, 20148 Milan, Italy; ^6^Department of Epidemiology and Preventive Medicine, University of Regensburg, Regensburg Franz-Josef-Strauß-Allee 11, 93053 Regensburg, Germany

## Abstract

Multiple sclerosis (MS) is characterized by a wide interpatient clinical variability and available biomarkers of disease severity still have suboptimal reliability. We aimed to assess immunological and MRI-derived measures of brain tissue damage in patients with different motor impairment degrees, for *in vivo* investigating the pathogenesis of MS-related disability. Twenty-two benign (B), 26 secondary progressive (SP), and 11 early, nondisabled relapsing-remitting (RR) MS patients and 37 healthy controls (HC) underwent conventional and diffusion tensor brain MRI and, as regards MS patients, immunophenotypic and functional analysis of stimulated peripheral blood mononuclear cells (PBMC). Corticospinal tract (CST) fractional anisotropy and grey matter volume were lower and CST diffusivity was higher in SPMS compared to RRMS and BMS patients. CD14+IL6+ and CD4+IL25+ cell percentages were higher in BMS than in SPMS patients. A multivariable model having EDSS as the dependent variable retained the following independent predictors: grey matter volume, CD14+IL6+ and CD4+IL25+ cell percentages. In patients without motor impairment after long-lasting MS, the grey matter and CST damage degree seem to remain as low as in the earlier disease stages and an immunological pattern suggestive of balanced pro- and anti-inflammatory activity is observed. MRI-derived and immunological measures might be used as complementary biomarkers of MS severity.

## 1. Introduction

A hallmark of multiple sclerosis (MS) is the wide intra- and interpatient phenotypic variability. Since the earliest descriptions of MS, it has been reported that the commonest clinical form, that is, the relapsing-remitting (RR) one, does often evolve into a disabling, secondary progressive (SP) stage within 15 years [1]. Conversely, a nondisabling course, named benign MS (BMS), can occur in patients with minimal motor impairment 15 years after the onset, even if they experience clinical relapses. Nonetheless, the actual existence of “truly” BMS is still a matter of debate, since several reports describe BMS patients accumulating disability and even converting to SP in a later phase of the disease [2, 3]. Therefore, different aspects of MS, including cognitive functioning, MRI features, and immunological markers, have been extensively investigated [4–6] with the aim to achieve a better knowledge of BMS versus SPMS and to identify possible biomarkers associated with a favorable disease course.

During the last 20 years, MRI techniques have broadened our knowledge on the mechanisms underlying disability accrual in MS [7, 8]. More recently, several MRI-based studies reported that a regional quantification of damage in clinically eloquent areas has a better correlation with disability than global measures [9, 10]. The analysis of immunological data is also of outmost importance to understand the pathogenesis of different MS clinical forms. No reliable laboratory markers of MS severity have been found yet, but several studies investigating cytokine levels and lymphocyte subsets from the peripheral blood of MS patients [11, 12] have shed some light on the balance between tissue damage and repair and on the different recruitment of the various components of the immune system according to the disease stage and phenotype.

Against this background, this cross-sectional study was conducted to obtain both laboratory parameters reflecting immune system functioning and MRI-derived markers of tissue damage from patients with BMS, SPMS, and early RRMS. The dual aim was to better investigate the potential of different biomarkers of MS severity and to increase our knowledge about the mechanisms related to the aforementioned clinical disease heterogeneity.

## 2. Material and Methods

### 2.1. Subjects

Patients with established MS [13] were recruited from the population attending the MS Center of the Fondazione Don Gnocchi. All patients had to be relapse- and steroid-free for at least 3 months. Complete neurological examination with expanded disability status scale (EDSS) score rating [14] was performed in all patients by a single neurologist. MS course had to be benign (disease duration ≥ 15 years, EDSS ≤ 3.0), early, nondisabling RR (disease duration ≤ 3 years, EDSS score ≤ 3.0) or SP [15]. Patients had to be free from acute or chronic infections. Healthy controls (HC) from a previous study [16] (group 1 HC) were used to prepare a corticospinal tract (CST) tractographic atlas. Other subjects with no history of neurological, cardiovascular, or metabolic disorders and a normal neurological examination, age-matched with MS patients (group 2 HC), were recruited as controls for the MRI evaluations. The study was approved by the local Ethics Committee and a written informed consent was obtained from all subjects prior to study entry.

### 2.2. MRI Acquisition

Brain MRI was acquired from all subjects using a 1.5 Tesla scanner (Siemens Magnetom Avanto, Erlangen, Germany), equipped with a 12-channel head coil. The following sequences were obtained: (1) dual-echo turbo spin echo (repetition time (TR) = 2650 ms, echo time (TE) = 28/113 ms; echo train length = 5; flip angle = 150°, 50 axial slices with a matrix size = 256 × 256, interpolated to 512 × 512, field of view (FOV) = 250 × 250 mm, slice thickness 2.5 mm); (2) 3D T1-weighted magnetization-prepared rapid acquisition gradient echo (MP-RAGE) (TR/TE/inversion time = 1900/3.37/1100 ms, flip angle = 15°, 176 axial slices, voxel size = 1 × 1 × 1 mm, 192 × 256 matrix); (3) diffusion-weighted single shot spin-echo sequence (TR/TE = 7100/94 ms, 50 axial slices, 128 × 96 matrix, FOV = 320 × 240 mm, slice thickness = 2.5 mm). The DTI protocol included two runs, each with 12 noncollinear diffusion gradients (*b* = 900 s/mm^2^) and 1 nondiffusion-weighted (*b* = 0 s/ mm^2^); (4) postcontrast (gadoteridol 0.1 mmol/Kg) T1-weighted (TR/TE = 650/44 ms, 44 axial slices, slice thickness 3 mm, 256 × 256 matrix, FOV = 250 × 250 mm). All slices were positioned to run parallel to a line that joins the most inferior-anterior and inferior-posterior parts of the corpus callosum.

### 2.3. MRI Data Analysis and Postprocessing

MS lesions were identified on proton density-weighted scans, using the corresponding T2-weighted images to increase confidence in lesion identification. Lesion segmentation and brain lesion volume (LV) computation were performed using the Jim software (Jim 5.0, Xinapse System, Leicester, UK). The presence and number of enhancing lesions on postcontrast T1-weighted images were also assessed.

3D T1-weighted images were processed with the cross-sectional version of the Structural Image Evaluation of Normalized Atrophy software (SIENAX) [17], with a mask image of the lesions (previously coregistered from T2 to T1 volume). The normalized volumes of grey matter (NGMV), cortical grey matter (NCV), white matter (WMV), and brain parenchyma (NBV) were measured. Diffusion-weighted images were preprocessed using FSL (http://www.fmrib.ox.ac.uk/fsl/), and then DT in every voxel and the DT-derived maps, that is, mean diffusivity (MD) and fractional anisotropy (FA), were estimated using Diffusion Toolkit software version 0.4.2 (http://www.trackvis.org/), following a process described elsewhere [16]. MS lesions (previously coregistered to the *b* = 0 volume) were masked out from the DT-derived maps. We created a left and right CST probabilistic atlas, as described in the literature [18], using TrackVis v0.4.3 (http://www.trackvis.org/) for the CST tractography of group 1 HC. With the probabilistic atlas and the lesion-masked DT-derived maps of every subject, the mean FA and MD of the normal-appearing CST were computed for MS patients and group 2 HC, using the method described in the literature [19].

### 2.4. Immunology

Blood samples were obtained from MS patients within 24 hours from MRI scan acquisition. Data from an historical control group of 40 age- and sex-matched healthy subjects were used to provide normal reference values. In order to identify immunological biomarkers, the functional differentiation of Myelin Basic Protein- (MBP-) stimulated CD4+ and CD14+ cell subsets was assessed using immunofluorescent staining protocol and flow-cytometry analysis [20]. The following immune cell subsets were analysed and categorised as markers of (a) proinflammatory response: CD4+IFN*γ*+, CD4+IL17+, CD14+IL6+, and CD14+IL12p35+ cells; (b) anti-inflammatory response: CD4+IL13+, CD4+ IL25+, CD14+IL10+ and CD14+TGF-*β*+ cells; (c) tissue repair: CD4+BDNF+ cells [20].

### 2.5. Statistical Analysis

Median and interquartile ranges were used according to skewness evaluated by the Kolmogorov-Smirnov test. Univariate correlations between clinical and paraclinical variables were assessed using the Spearman correlation coefficient with Fisher's Z transformation [21].

Paraclinical variables were normalized using the Blom's transformation [22] and compared by group using a generalized linear model (GLM) analysis of variance model corrected for age, gender, and therapy as dummy variables. To account for unequal number of observations, multiple comparisons between groups were performed by the Tukey-Kramer [23] method. Least squares means and 95% confidence limits were obtained by retrotransformation.

To investigate which MRI and immunological variables were independent predictors of patients' EDSS, a variable selection method based on the GLM approach was used.

To assess whether immunological variables were significantly different between treated and untreated patients, we used GLM analysis of variance corrected for age and gender.

All statistical tests were two-tailed and Bonferroni correction for multiple comparisons was applied to all univariate tests.

Statistical evaluations were performed using SAS statistical software v. 9.2.

## 3. Results

Fifty-nine patients (22 with BMS, 11 with RRMS and 26 with SPMS) and 37 HC were studied (Table 1). No differences between MS patients and HC were found for gender (*χ* ^2^ = 1.59, *P* = 0.45) and age (*P* = 0.74). At* post hoc* comparisons, an age difference (*P* = 0.024) was found between RRMS and SPMS patients. No significant difference between SPMS and BMS patients was found for age and disease duration, which was, for the inclusion criteria, lower (*P* < 0.0001) for RRMS patients. At the time of the study, 22 (37%) patients were being treated with disease-modifying drugs: 13 with interferon *β*-1a, 1 with interferon *β*-1b, 6 with glatiramer acetate, and 2 with azathioprine. Disease phenotypes in these patients were: BMS in 8 (36%), 6 treated with interferon *β*-1a and 2 with glatiramer acetate; RRMS in 8 (73%), 5 treated with interferon *β*-1a, 1 treated with interferon *β*-1b, 1 with glatiramer acetate, and 1 with azathioprine; SPMS in 6 (23%), 2 treated with interferon *β*-1a, 3 with glatiramer acetate, and 1 with azathioprine.

### 3.1. MRI Findings

Contrast-enhancing lesions were found in 1 BMS patient (4%), 4 RRMS patients (36%), and 15 SPMS patients (58%), that is, in a total of 20 patients (34%) in the whole MS cohort. Tables 2 and 3 report the values of MRI-derived measures in the whole cohort and in the different subjects' subgroups. All MRI variables were normally distributed within each subgroup.

LV was the highest in SPMS patients, but there were no significant differences between patients' subgroups. All brain tissue volumes were lower in MS patients than in HC. All brain tissue volumes were not statistically different between BMS and RRMS, while NBV and NGMV were lower in SPMS than in BMS and RRMS patients.

In both HC and MS patients, a difference (*P* < 0.0001) was found between left and right CST FA and MD values, with FA being lower and MD higher in the right tract. Average left and right CST FA were lower in MS patients than in HC and heterogeneous between patient subgroups. At* post hoc* comparisons, SPMS patients had lower FA and higher MD values than both BMS and RRMS patients, but no differences were found between the latter subgroups.

### 3.2. Immunological Findings

Cytokine expression was not observed in cell cultures incubated with nonantigenic peptides, according to published results (data not shown) [24]. Tables 4 and 5 report the values of individual cell subset percentages in the study subjects.

Regarding markers of proinflammatory response, CD4+IL17+ cell percentage was the only one that significantly increased in MS patients compared to the normal reference values. CD14+IL6+ cells were higher in MS patients than in the normal control group too, but the difference did not reach statistical significance. However, the percentage of CD14+IL6+ cells was higher in BMS than in SPMS (*P* < 0.0001) and RRMS (*P* = 0.004) patients (Table 5).

Regarding anti-inflammatory markers, CD4+IL13+ and CD4+IL25+ cell percentages were significantly increased in MS patients compared to the normal reference values. The analysis of between-patient group differences showed that CD4+IL25+ cell percentage was lower in SPMS than in RRMS (*P* < 0.0001) and BMS patients (*P* < 0.0001) (Table 5).

CD4+BDNF+ cell percentage was significantly increased in MS patients compared to the normal reference values, but did not differ between MS phenotypes.

No cell subset percentages were significantly different between treated and untreated patients (data not shown).

### 3.3. Clinical Correlations

Univariate correlations between patients' EDSS and paraclinical variables are reported in Table 6. With the exception of WMV and left CST MD, all MRI metrics showed a significant relationship with disability levels, with the highest being with NGMV. Higher disability levels were associated with lower CD14+IL6+ and CD4+IL25+ cell percentages, with the strongest correlation being the one with CD4+IL25+.

The final multivariable model having EDSS as the dependent variable retained the following independent predictors: NGMV (beta = −0.01, SE = 0.003, *P* < 0.0001), CD4+IL25+ cell percentage (beta = −3.5, SE = 1.29, *P* = 0.01), and CD14+IL6+ cell percentage (beta = −0.04, SE = 0.02, *P* = 0.07) (global *R* ^2^ = 0.57, intercept: beta = 14,5, SE = 1,89, *P* < 0.0001).

## 4. Discussion

The identification of biomarkers of MS severity and a better understanding of its pathogenesis remain issues of outmost importance to improve our workup of the disease as regards the search for novel treatment strategies and the assessment of their efficacy. To the best of our knowledge, this is the first* in vivo* study where MRI-derived and immunological metrics have been investigated in combination to readdress these aims.

In our sample of patients with levels of disability ranging from absent to severe motor impairment different clinical phenotypes are represented, that is, RR, SP, and benign MS. GM volumes were the lowest in SPMS, while there was no significant difference between RRMS and BMS, although the latter subgroup had a disease duration comparable to that of SPMS. These findings indicate that GM volume is the MRI parameter with the strongest ability to discriminate between MS patients with high versus low levels of disability, independently of the disease duration. The two main mechanisms implicated in the pathogenesis of GM damage are cortical demyelination, resulting in lesions detectable with* ad hoc* MRI sequences, and neurodegeneration. The latter feature, reflected by measures of tissue atrophy, has been described in all MS phenotypes [8, 25], including BMS. Indeed, our study did not include cognitive assessment, which enables us to detect the presence of “occult” disease-related disability in about one-third of BMS patients [26]. Despite this limitation, leading to a possible overestimation of the “benignity” of this subgroup, measures of GM damage did not significantly differ between BMS and RRMS patients with a sixfold shorter disease duration. This indicates that GM pathology, although present, is much less severe and destructive in BMS, leading to a lower disability and a slower progression of the disease. As a consequence, reparative mechanisms might have the possibility to act more effectively and limit the clinical impact of tissue damage. A complementary explanation for the milder disease course shown by BMS might be the different topographical distribution of brain damage, with relative sparing of clinically eloquent areas [4, 27, 28]. Against this background, considering that the definition of BMS relies on EDSS scoring, which is mainly determined by motor disability, the comparison of CST-related measures between BMS and the other phenotypes is of particular interest. CST FA was altered in MS with respect to healthy controls, and SPMS patients showed a more severe CST involvement than BMS and RRMS. CST FA and MD also correlated with the level of disability, as previously reported [29]. Once again, BMS did not differ from early RRMS in any of these measures of CST damage. Considering that FA is mainly related to axonal damage, these findings strengthen the hypothesis that neurodegeneration in BMS is less destructive or occur more slowly, thus allowing patients to benefit from reparative or compensatory processes. In contrast with a previous study [30], reporting no difference between BMS and HC in CST FA, our BMS cohort had a significantly lower CST FA than HC (data not shown). One possible explanation is that BMS patients belonging to that study had a slightly shorter disease duration and cognitive impairment was an exclusion criterion, whereas our patients were not cognitively assessed, thereby allowing for the inclusion of less “benign” cases. Consistently with the results of between-group comparisons, the strongest correlation with patients' EDSS scores was achieved by NGMV values, but there was also a moderate relationship with DT-derived measures of CST integrity.

Immunological data can be viewed as complementary to MRI, because they give information on the balance between inflammatory/anti-inflammatory processes and repair mechanisms. The cytokine network is very complex, and very often the production/inhibition of some elements is strictly intertwined with many other components of the immune system. To further complicate the picture, some elements, such as TGF*β*, can play a dual role, stimulating the production of Th17 proinflammatory cells and cytokines (IL17) and, on the other hand, protecting against autoimmune diseases through tolerance induction. In addition, more than one-third of our patients were being treated with disease-modifying therapies, which have a great impact on several components of the immune system [31]. This, together with the cross-sectional and observational nature of the study, does not allow us to draw any conclusions on the possible pathogenetic roles of the cytokine analyzed.

We observed an increase of CD4+IL17+ cells in all MS subtypes, confirming published data on their role in MS pathogenesis in different disease stages [11]. The presence of an important inflammatory component in SPMS, in which neurodegeneration is thought to prevail [32], is confirmed by the relatively high percentage of patients showing contrast-enhancing MRI lesions. This result is similar to the high percentage of active MRI (53%) found in a previous study [33] of 60 untreated SPMS patients and might be explained by the low number of treated SPMS subjects (8 of 26) in our sample. Indeed, interferon-beta trials showed that treatment was effective in SPMS only when relapses were still superimposed to disability progression [34], confirming that a variable balance between inflammation and neurodegeneration can be present in patients with this disease phenotype and that both these components have an impact on disability accrual.

CD14+IL6+ cell percentage was also higher in MS patients than in HC, although the difference did not reach statistical significance. This finding is consistent with the elevated levels of CD4+IL17+, considering that IL6 is responsible for the amplification of the inflammatory response through several mechanisms including Th17 proliferation and recruitment. Interestingly, CD14+IL6+ cells were significantly higher in BMS than in RRMS and SPMS. On the other hand, recent data [35] indicate that IL6-driven inflammation is evident in progressive MS and in motor neuron disorders, which are both characterised by a disabling course. An explanation for our findings might be that the inflammatory response detected in BMS may, to some extent, exert beneficial effects for tissue preservation and be promoted to counterbalance tissue damage. Some authors have indeed speculated on the possible positive role of inflammation in MS, hypothesizing that inflammatory reactions might promote repair and remyelination and confer neuroprotection [36, 37]. In SPMS, the inflammatory pattern seems to be more different than in the earlier stages of the disease, since in the periphery there is a predominance of Th2, anti-inflammatory cells [12] and inflammation is thought to be mainly sustained by a humoral component sequestered inside the CNS [38, 39].

Anti-inflammatory cytokine-expressing lymphocyte levels were elevated in MS patients compared to HC, possibly and at least partially because of the immunomodulatory effects of ongoing treatments. However, it is conceivable that the inflammatory reaction associated with MS evokes an anti-inflammatory, Th2-driven response that is maintained over the course of the disease. It is worth noting that CD4+IL25+ cell percentage was significantly lower in SPMS than in both BMS and RRMS patients. In addition, CD4+IL25+ cell percentage was not significantly different between HC and SPMS. This finding might be explained taking into consideration the aforementioned compartmentalized humoral immune response described in SPMS patients, associated with a less pronounced involvement of peripheral immune system. It is worth noting, however, that low levels of CD4+IL25+ cells were overall associated with increasing patients' disability and with MRI features suggestive of pronounced tissue damage (i.e., more severe brain atrophy and CST abnormalities), indicating that a decrease of anti-inflammatory response could indeed be responsible for a less effective disease control. At any rate, one should always bear in mind the difficulties to interpret immunological findings obtained* in vivo* from peripheral blood samples, which can be influenced by biological factors independent of MS course.

The results of the multivariable analysis are consistent with findings from group comparisons between patients with different MS phenotypes and indicate that increasing EDSS scores are explained by more severe GM damage and by a decrease of both proinflammatory and anti-inflammatory cytokine-producing lymphocytes in the peripheral blood.

## 5. Conclusions

Both MRI and immunological parameters were retained in a multivariable model able to predict patients' locomotor disability on a cross-sectional basis. Given the intrinsic variability of immunological and MRI biomarkers, however, it could be worth assessing them in an independent population to confirm our results. In addition, this calls for longitudinal studies aimed at evaluating whether a combination of these metrics could enable us to early identify MS patients with a favourable medium- to long-term prognosis.

## Figures and Tables

**Table 1:** Demographic and clinical characteristics of the study subjects.

	HC_lab_	HC	All MS	BMS	RRMS	SPMS
Number	40	37	59	22	11	26
Mean age (SD) (years)	44.2 (10.7)	45.7 (14.4)	46.2 (9.8)	46.5 (7.2)	37.5 (9.5)	49.6 (9.9)
Men/women	12/28	15/22	21/38	9/13	5/6	7/19
Mean disease duration (SD) (years)		—	18.0 (9.6)	20.9 (4.8)	2.1 (0.7)	22.4 (7.6)
Median EDSS (range)		—	3.0 (0.0–7.5)	1.5 (0.0–3.0)	1.5 (0.0–3.0)	6.0 (4.0–7.5)

HC_lab_: historical healthy controls for immunology; HC: healthy controls recruited for magnetic resonance measures; MS: multiple sclerosis; BMS: benign MS; relapsing-remitting MS; secondary progressive MS.

**Table 2:** Conventional and DT MRI findings from HC and all MS patients.

	HC (*n* = 37)	All MS (*n* = 59)	HC versus MS
LV	—	16.5 (15.5)	—
NBV	1531.3 (78.1)	1462.2 (91.2)	<0.001
NCV	624.0 (48.4)	577.8 (74.6)	<0.0001
NGMV	790.6 (55.2)	722.9 (95.4)	<0.0001
NWMV	737.6 (36.7)	739.2 (39.2)	<0.001
Left CST FA	0.515 (0.038)	0.490 (0.050)	0.016
Left CST MD	0.727 (0.029)	0.736 (0.051)	ns
Right CST FA	0.496 (0.024)	0.459 (0.039)	<0.001
Right CST MD	0.749 (0.026)	0.762 (0.053)	ns

Data are reported as mean (standard deviations). *P* values from group comparisons (*t*-test) are reported. Lesion and tissue volumes are expressed in cc, MD is expressed in 10^−3^ mm^2^ s^−1^, and FA is a dimensionless index.

LV: total brain lesion volume; NBV: normalized brain volume; NCV: normalized cortical grey matter volume; NGMV: normalized grey matter volume; NWMV: normalized white matter volume; CST: corticospinal tract; MD: mean diffusivity; FA: fractional anisotropy; ns: not significant.

See the text for other abbreviations and statistical analysis details.

**Table 3:** Conventional and DT MRI findings from patients with different MS phenotypes.

	BMS			RRMS			SPMS			BMS versus RRMS	BMS versus SPMS	SPMS versus RRMS
	LS mean	95% CL		LS mean	95% CL		LS mean	95% CL	
LV	10.6	4.5	16.6	6.8	−2.3	16.0	28.4	21.9	35.0	ns	ns	ns
NBV	1483.3	1452.4	1514.2	1564.3	1517.3	1611.3	1386.1	1352.6	1419.6	ns	0.001	0.003
NCV	605.3	579.0	631.6	642.8	602.8	682.7	516.9	488.4	545.4	ns	ns	ns
NGMV	759.1	726.1	792.2	809.9	759.7	860.2	644.1	608.3	679.9	ns	<0.001	0.0001
NWMV	724.1	706.6	741.6	754.3	727.7	781.0	742.0	723.0	761.0	ns	ns	ns
Left CST FA	0.505	0.486	0.524	0.536	0.506	0.565	0.455	0.434	0.476	ns	<0.001	0.002
Left CST MD	0.720	0.698	0.742	0.713	0.680	0.746	0.765	0.741	0.789	ns	<0.001	<0.0001
Right CST FA	0.473	0.458	0.488	0.496	0.473	0.518	0.432	0.416	0.448	ns	<0.0001	<0.0001
Right CST MD	0.746	0.725	0.767	0.723	0.691	0.756	0.794	0.771	0.817	ns	<0.0001	<0.0001

Data are reported as least squares (LS) means and 95% confidence limits (CL). Lesion and tissue volumes are expressed in cc, MD is expressed in mm^2^s^−1^ × 10^−3^, and FA is a dimensionless index. Between-group comparison *P* values were corrected with Tukey-Kramer and the following variables were taken as covariates: therapy (dichotomic variable: yes/no), age, and gender.

LV: total brain lesion volume; NBV: normalized brain volume; NCV: normalized cortical grey matter volume; NGMV: normalized grey matter volume; NWMV: normalized white matter volume; CST: corticospinal tract; MD: mean diffusivity; FA: fractional anisotropy; ns: not significant.

See the text for other abbreviations and statistical analysis details.

**Table 4:** Percentages of MBP-stimulated cytokine producing blood cells from historical controls and all MS patients.

Cell type	Historical controls	All MS	All MS versus historical controls
CD4+IL17+	0.0 (0-0)	0.04 (0–0.08)	<0.0001
CD4+IFN*γ*+	0.01 (0–0.08)	0.01 (0–0.03)	ns
CD14+IL12p35+	0.0 (0-1)	0.13 (0–2.01)	ns
CD14+IL6+	0.0 (0-1)	1.9 (0–5.5)	ns
CD4+IL13+	0.05 (0–0.1)	0.12 (0.01–0.16)	0.003
CD4+IL25+	0.13 (0–0.15)	0.33 (0.1–0.5)	<0.0001
CD14+IL10+	12.0 (0.03–26.8)	12.1 (0.17–21.3)	ns
CD14+TGF*β*+	0.0 (0-1)	1.65 (0–6.25)	ns
CD4+BDNF+	0.07 (0–0.43)	0.72 (0–2.8)	<0.0001

Data are reported as median values (interquartile ranges).

*P* values from group comparisons (*t*-test) are reported; ns: not significant.

**Table 5:** Percentages of MBP-stimulated cytokine producing blood cells from patients with different MS phenotypes.

Cell type	BMS			RRMS			SPMS			BMS versus RRMS	BMS versus SPMS	SPMS versus RRMS
	LS mean	95% CL		LS mean	95% CL		LS mean	95% CL	
CD4+IL17+	0.07	−0.02	0.21	0.09	−0.07	0.24	0.12	0.02	0.23	ns	ns	ns
CD4+IFN*γ*+	0.02	−0.14	0.17	0.16	−0.09	0.42	0.13	−0.08	0.34	ns	ns	ns
CD14+IL12p35+	2.02	0.90	3.13	0.93	−0.80	2.65	1.17	−0.08	2.42	ns	ns	ns
CD14+IL6+	15.24	10.53	19.96	1.72	−4.82	8.27	0.64	−4.03	5.32	0.004	<0.0001	ns
CD4+IL13+	0.16	0.13	0.20	0.12	0.06	0.17	0.11	0.07	0.15	ns	ns	ns
CD4+IL25+	0.48	0.40	0.56	0.60	0.48	0.72	0.18	0.08	0.27	ns	<0.0001	<0.0001
CD14+IL10+	13.14	4.78	21.50	11.80	−0.05	23.64	20.32	11.32	29.32	ns	ns	ns
CD14+TGF*β*+	2.02	−0.63	4.66	5.52	1.66	9.38	4.83	2.03	7.62	ns	ns	ns
CD4+BDNF+	1.77	−2.31	5.84	9.76	2.81	16.71	0.61	−3.68	4.90	ns	ns	ns

Data are reported as least squares (LS) means and 95% confidence limits. Between-group comparison *P* values were corrected with Tukey-Kramer and the following variables were taken as covariates: therapy (dichotomic variable: yes/no), age, and gender; ns: not significant.

**Table 6:** Univariate correlations between patients' EDSS, MRI-derived, and immunological variables.

	SCC	95% CL		*P* value
MRI metrics				
LV	0.420	0.151	0.612	0.001
NCV	−0.555	−0.709	−0.342	<0.0001
NGMV	−0.593	−0.736	−0.391	<0.0001
NWMV	0.095	−0.168	0.344	ns
NBV	−0.576	−0.724	−0.368	<0.0001
Left CST FA	−0.401	−0.595	−0.156	0.0016
Left CST MD	0.310	0.054	0.524	ns
Right CST FA	−0.469	−0.646	−0.236	0.0002
Right CST MD	0.365	0.114	0.567	0.0046
Cell percentages				
CD4+IL17+	0.062	−0.210	0.324	ns
CD4+IFN*γ*+	−0.172	−0.480	0.179	ns
CD14+IL12p35+	0.054	−0.219	0.319	ns
CD14+IL6+	−0.418	−0.621	−0.153	0.0023
CD4+IL13+	−0.116	−0.386	0.175	ns
CD4+IL25+	−0.611	−0.760	−0.390	<0.0001
CD14+IL10+	0.095	−0.184	0.358	ns
CD14+TGF*β*+	0.127	−0.155	0.388	ns
CD4+BDNF+	−0.057	−0.369	0.268	ns

Values are corrected for therapy (dichotomic variable), age and gender.

SCC: Spearman rank correlation coefficients; CL: confidence limits; LV: total brain lesion volume; NBV: normalized brain volume; NCV: normalized cortical grey matter volume; NGMV: normalized grey matter volume; WMV: normalized white matter volume; CST: corticospinal tract; MD: mean diffusivity; FA: fractional anisotropy. See the text for other abbreviations and statistical analysis details.
